# Different Plant Sporopollenin Exine Capsules and Their Multifunctional Usage

**DOI:** 10.1021/acsabm.2c00071

**Published:** 2022-02-24

**Authors:** Funda Ersoy Atalay, Ayse Asiye Culum, Harun Kaya, Gunay Gokturk, Emel Yigit

**Affiliations:** † Department of Physics, The Faculty of Science and Arts, Inonu University, 44280 Malatya, Turkey; ‡ Department of Medical Services and Techniques, Vocational School of Health Services, Malatya Turgut Ozal University, 44210 Malatya, Turkey; § Faculty of Engineering and Natural Sciences, Malatya Turgut Ozal University, 44210 Malatya, Turkey; ∥ Department of Biology, The Faculty of Science and Arts, Inonu University, 44280 Malatya, Turkey

**Keywords:** sporopollenin exine capsule, drug delivery, biotemplate, pollen, porous, supercapacitor, electrode

## Abstract

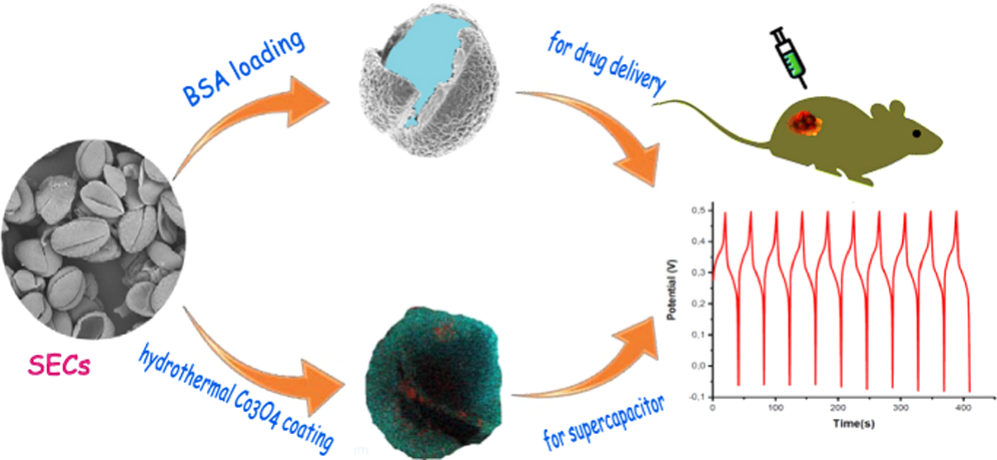

Sporopollenin exine capsules (SECs) are highly resistant to heat and various acids and bases. They are also cheap, highly porous, eco-friendly polymer biomaterials with stable microencapsulation capacity. Due to their strong and uniquely shaped exine layers, they can allow growth on metal oxide materials, as a biotemplate for use in different applications. In this study, first, a single SEC extraction method was applied to three different pollens from *Pinus*, *Fraxinus excelsior*, and *Tilia*. Scanning electron microscopy (SEM), Brunauer–Emmett–Teller (BET) analysis, and thermogravimetric/differential thermal analysis (TGA/DTA) measurements both before and after the extraction process were performed to observe changes in surface area, morphology, porous structure, and degradation properties. The protein content and removal were analyzed by elemental and spectrophotometric analyses. Then, SECs were loaded by passive and centrifuge loading for drug delivery, and the loading capacities were analyzed by Fourier transform infrared spectroscopy and spectrophotometry. The method was successful in opening the pores and maintaining the structural integrity of SECs. It was determined that the morphology and porosity affected the encapsulation efficiency. According to the loading capacities, *Tilia* SECs were the most efficient SECs for both loading methods. In addition, three different SECs were hydrothermally coated with cobalt and then heat-treated to obtain a metal oxide structure. A CO_3_O_4_ supercapacitor electrode constructed using CO_3_O_4_-*F. excelsior* SEC powder had the best surface area parameters. The electrode showed a maximum specific capacity of 473 F/g for over 3000 continuous cycles of galvanostatic charge–discharge (GCD).

## 1 Introduction

Pollen is the structure of the plant that carries the male gamete to the female gamete for fertilization.^1^ It protects male gametes from environmental influences during transport owing to its stable and inert structure.^2^ The chemical structure, surface properties, and dimensions of pollen vary considerably among the species.^3−5^ The pollen grain’s stable structure, known as the sporopollenin exine capsule (SEC), is obtained from the exoskeleton shells (exine). Their size ranges from 4 μm (*Myosotis*, forget-me-not) to 250 μm (*Cuburbita*, pumpkin). The SEC is a highly cross-linked organic polymer. There are many channels in the exine wall with an average thickness of 2 μm that make the structure porous. Therefore, both the inner and outer surfaces of the exine are suitable for possible binding, which is useful in multifunctional technological applications.^6^ Being a chemically highly inert and ubiquitous biopolymer,^7^ the SEC is suitable for applications such as medical imaging, the delivery of pharmaceuticals and other active substances,^6^ heavy metal removal, catalyst support, and taste suppression.^2^ The loading efficiencies for drug delivery applications were investigated in several SECs such as pine,^8^ date palm,^9^*Corylus avellane*,^10^ dandelion,^11^ plane tree,^12^ and *Lycopodium clavatum*.^13,14^

Pollen grains are renewable materials and abundant in nature. SECs extracted from pollen grains have advantages over synthetic encapsulants such as a large inner cavity, uniformness in size and shape, mucoadhesion, biocompatibility, resistance to heat and harsh environments, UV and oxidation protection, and properties of surfaces.^10,13^ In addition to these properties, they are able to pass the gut wall.^15^ On the other hand, there are a limited number of studies in the literature using plant pollen for microencapsulation. Barrier et al. investigated the viability of plant spore exine capsules for microencapsulation in *L. clavatum* SECs.^13^ To see which substances pass through the nm thick channels found in the SECs, encapsulation of different materials has been tried such as dyes of different polarities, oils, fats, and resins, and it has been proven that they pass through these channels. Cod liver oil, sHRP, and alkaline phosphatase (ALP) enzymes were encapsulated in SECs and were recovered with only little loss of enzyme activity, without damage to other materials.^13^ Hamad et al. have achieved to encapsulate living yeast cells in a *L. clavatum* SEC.^15^ It has also been shown that successful results can be obtained when used in oral vaccination.^16^ All of these studies prove that SECs are nontoxic and nonallergic natural materials that can be used widely in drug delivery, cosmetics, and the food industry.

Several studies have been performed to increase the energy storage capacity of supercapacitors for use in the commercial market.^17^ Some organic structures that cannot be synthesized chemically due to the unique nanostructured surface properties can be used in the design of supercapacitor electrodes.^18^ The most important factors affecting the electrochemical performance of supercapacitor electrodes are the specific surface area, pore shape and structure, pore shape distribution, surface functionality, and electrical conductivity of the active material used in the electrode design.^17^ Some studies have been reported in which supercapacitor electrodes were produced with the use of biological materials such as algae, fungi, viruses, and pollen.^19,20^ Among these structures, pollen attracts attention in terms of being obtainable from nature directly, in the easiest and cheapest way, with unique surface structures and surface diversity. Supercapacitor electrodes were usually made of carbonized pollen (camellia,^21,22^ lotus,^22,23^ peony,^22^ oilseed rape,^22^ or pine^24^) as an active carbon source. However, studies in which metal oxides were formed with the use of a pollen template and then used as electrode active material are rare.

Among many transition metal oxide materials such as RuO_2_, ZnO, MnO_2_, and SnO_2_ as the electrode active material in supercapacitors, Co_3_O_4_ stands out because of its environmental compatibility and its easy and economical production methods.^25−28^ Because of the very high theoretical capacity of Co_3_O_4_ (3560 F/g), it has been studied intensively recently. However, due to slow electrode kinetics and rapid corrosion in the electrolyte, a rapid decrease in capacitance is observed.^25−28^ To enhance the electrochemical performance, composites were produced by combining Co_3_O_4_ with other metal oxides, polymers, and carbon structures.^25−31^

Until now, Co_3_O_4_ materials in the form of nanoparticles, nanorods, nanowires, nanobelts, and nanotubes have been produced by many different methods.^25−31^ Especially in materials produced in one-dimensional form (1D), the main problem is the desire for nanoparticle agglomeration.

Iqbal et al.^31^ have reported that the electrical conductivity and electrochemical stability of Co_3_O_4_ NPs can be improved by combining them with porous carbon nanofibers (CNFs) to form a hybrid supercapacitor. A 1D Co_3_O_4_@CNF electrode produced in combination with one-dimensional CNFs showed a specific capacitance of 80 F/g at 1A/g.^32^ It has been reported that electrodes formed from Co_3_O_4_ nanoparticles combined with a two-dimensional (2D) reduced graphene material (rGO) have better electrochemical storage capacity, due to effective ion transfer and reduced agglomeration.^27,33^ The specific capacitance was observed to be 278 F/g in the Co_3_O_4_ nanocube intercalated with an rGO matrix supercapacitor.^33^ Xiang et al.^27^ also reported that the rGO–Co_3_O_4_ composite electrode has shown the maximum specific capacitance (458 F/g), high rate capability, and high energy density and power density, which was attributed to the combination of Co_3_O_4_ with the high electronic conductivity of the rGO sheets.

Interest in pollen is increasing day by day due to its unique nanostructured surface properties and the fact that it is an organic material.^34^ Several SEC extraction methods have been developed for *L. clavatum*;^13,35,36^ however, these methods are inefficient for different pollen species.^34^ In this work, a single SEC extraction method was developed for pollen grains of three plant species including *Pinus*, *Fraxinus excelsior*, and *Tilia*. Pollen microcapsules were investigated comparatively according to their suitability for nanostructured particle production. Additionally, the loading capacities of SECs were determined by passive and centrifuge loading for drug delivery.

Furthermore, *F. excelsior* SECs were coated with Co_3_O_4_ hydrothermally and used as active materials for designing supercapacitor electrodes. The use of hollow three-dimensional (3D) SEC microspheres is proposed here as a solution to the problem of agglomeration observed in 1D and 2D materials and the accompanying slow electron transfer, as well as less interaction between the electrolyte and active material. In this study, Co_3_O_4_ was hydrothermally grown on 3D hollow SECs with a high surface area/volume ratio. 3D Co_3_O_4_ microspheres were used as the supercapacitor’s active material.

## 2 Materials and Methods

### 2.1 Pollen Harvesting and Isolation

*Pinus* (pine), *F. excelsior* (ash tree), and *Tilia* (linden) pollen grains were investigated in this study. The male flowers of *F. excelsior*, the flowers of *Tilia*, and the pollen grains of *Pinus* were harvested from the campus of Inonu University in the spring.

*Pinus* pollen is the easiest pollen to collect. The pollen grains were harvested by shaking male cones. It was sieved first with a coarse sieve and then with 106 μm mesh sieves for the elimination of plant debris.

*F. excelsior* male flowers were dried by laying on blotting paper under sunlight. After drying, flowers were ground in an automatic mortar (Retsch RM 100) with a 6 mm mortar height for 20 min to obtain pollen in closed anthers. The ground samples were first sieved through a coarse sieve, then sieves with 212, 106, 45, and 38 μm mesh, respectively.

*Tilia* was collected with its leaves; after separation from the leaves, the flowering parts were dried by laying on blotting paper under sunlight. Then, the flowers were pounded in an automatic mortar, and sieved first with a coarse sieve and then with a 45 μm mesh sieve.

### 2.2 Extraction of SECs

The extraction method was similar to that of Mundargi et al.^14^ but partially modified. SECs were extracted in a two-step procedure including defatting and acidolysis. Twenty grams of pollen was suspended in 200 mL of acetone in a 500 mL round-bottom flask for defatting. The flask was placed in an ultrasonic bath at (Elma Transsonic TI-H-5) 60 °C under reflux for 30 min and vigorously stirred on a magnetic stirrer at 60 °C for 30 min. The duration of the procedure significantly decreased using an ultrasonic bath. The defatted pollen was separated into 50 mL plastic tubes and centrifuged at 9000 rpm, 4 °C for 15 min. After discarding the supernatant, the precipitate was washed twice with Milli-Q water and then twice with ethanol at 9000 rpm, 4 °C for 15 min.

Several acids such as hydrochloric acid, phosphoric acid, and sulfuric acid are used for the acidolysis step of SEC extraction. Among these, phosphoric acid provides clean and less damaged SECs.^8,14,34,37−39^ Therefore, phosphoric acid usage was preferred for its advantages in this study. For the acidolysis process, the precipitate was suspended in 200 mL of orthophosphoric acid in a 500 mL round-bottom flask. The flask was placed in the ultrasonic bath at 70 °C under reflux for 30 min, then stirred on a magnetic stirrer at 70 °C for 30 min. After the acidolysis process, the above centrifugation and washing processes were repeated exactly. Finally, SECs were dried at 60 °C for 16 h. Hereinafter, the material obtained after orthophosphoric acid treatment, namely, the sporopollenin exine capsule, will be referred to as SEC in short.

### 2.3 Scanning Electron Microscopy (SEM)

Raw pollen, acetone-treated pollen, and SECs were placed on clean Si substrates and then coated with 3 nm thick gold (Emitech K550x sputter coater, Quorum Technologies). Before and after the extraction, the morphological changes and pollen diameters of raw pollen, acetone-treated pollen, and SECs were obtained from SEM images captured with an acceleration voltage of 10.00 kV (FEI Nova NanoSEM 450).

### 2.4 Brunauer–Emmett–Teller Analysis (BET)

The surface areas, pore volumes, and pore diameters of raw pollen, acetone-treated pollen, and orthophosphoric acid-treated pollen (or SECs) were calculated from the N_2_ adsorption isotherms obtained in the 0.00 and 1.00 p/p° relative pressure range, using a Micromeritics Gemini VII 2390 BET analyzer.

### 2.5 Thermogravimetric/Differential Thermal Analysis (TGA/DTA)

Degradation properties and mass losses of raw pollen, SECs, and cobalt-coated SECs according to the increasing temperature and variations in energy were analyzed by thermogravimetric analysis (Shimadzu 50 thermogravimetric analyzer, Kyoto, Japan) and differential thermal analysis (Shimadzu 50 differential thermal analyzer, Kyoto, Japan). The samples were heated at a rate of 10 °C/min and up to 1000 °C in a N_2_ atmosphere.

### 2.6 Protein Quantification

Proteins are the biopolymers of amino acid residues. There are 20 main amino acids in natural proteins. An amino acid consists of a carboxyl group, amino group, and R group that differs in each amino acid, bonded to the same carbon. The R group is grouped as nonpolar aliphatic, aromatic, polar uncharged, positively charged, and negatively charged. The aromatic amino acids (phenylalanine, tyrosine, and tryptophan) show maximum absorbance at 280 nm. This characteristic strong absorbance of light is exploited by researchers in the characterization of proteins.^40^ Tryptophan is the most abundant amino acid among the aromatic amino acids in the cytoplasm and membrane proteins, therefore, it is the dominant source of UV absorbance at ∼280 nm.^41^ Prabhakar,^8^ Mundargi,^14,42,43^ and Fan^11^ have determined the protein amount in the sporopollenin exine capsule (SEC) by measuring the absorbance at 280 nm. That is why the supernatant was collected after each extraction step for protein quantification. Protein quantification was detected by measuring the absorbance at 280 nm (BioTec Epoch).

### 2.7 Elemental Analysis

The C, H, and N compositions of raw pollens and SECs were analyzed with a LECO CHNS-932. The percent of protein was calculated by multiplying the percent of nitrogen with a Kjeldhal conversion factor of 6.25.^44^

### 2.8 Bovine Serum Albumin (BSA) Loading

For this, 100 mg of SECs was vortexed for 10 min after adding 13% BSA, for the passive and centrifuge loading methods. Samples were shaken for 2 h on a heat block at room temperature at 500 rpm for passive loading and centrifuged for 30 min at 10 000 rpm at 4 °C for centrifugal loading. Then, the samples were centrifuged at 12 000 rpm for 3 min at 4 °C. The supernatant was discarded. The pellet was washed three times with 750 μL of water. The loaded SECs were frozen at −20 °C for 24 h. The samples were dried for 24 h at room temperature.

The amount of loaded BSA was determined after the ultrasonic bath step. Ten milligrams of loaded SECs were suspended in 1.4 mL of PBS buffer and vortexed for 5 min, and then placed in an ultrasonic bath for 30 s. Then, the samples were centrifuged at 15 000 rpm for 5 min. The supernatant was filtered through a 0.45 μm polyethersulfone (PES) syringe filter (Isolab) and the quantity of BSA was analyzed by spectrophotometry (BioTec Epoch). The BSA-loading content and encapsulation efficiency were calculated using the following equations^11^
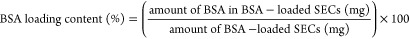
1
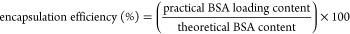
2

### 2.9 Fourier Transform Infrared Spectroscopy (FTIR)

BSA, SECs, and loaded SECs were analyzed between 4000 and 400 cm^–1^ infrared spectra for determining chemical interactions (Perkin-Elmer, Spectrum One FTIR spectrometer).

### 2.10 Hydrothermal Co_3_O_4_ Coating and Supercapacitor Electrode Preparation

Briefly, 0.3 g of SECs was mixed with 25 mM Co(NO_3_)_2_ and 25 mM ethanol for 1 h on a magnetic stirrer. It was then transferred to a 100 mL Teflon-lined sealed steel hydrothermal reaction vessel and subjected to hydrothermal treatment at 150 °C for 4 h. The resulting precipitate was dried in a vacuum oven at 60 °C for 12 h. Then, heat treatment was applied in the air at 360 °C for 2 h.

The resulting material was used as an active material in the production of supercapacitor electrodes. For this, a cobalt-containing active material (75%), acetylene black (15%), and poly(vinylidene fluoride) (PVDF) (10%) were mixed thoroughly in a Zr_2_O_3_ agate mortar for 45 min. Then, 400 μL of *N*-methyl-2-pyrrolidone (NMP) was added to this homogeneous mixture, and the resulting slurry was spread on cleaned Ni foam and dried in a vacuum oven at 60 °C. Then, the working electrode was obtained by pressing it under a pressure of 5 MPa.

### 2.11 Electrochemical Measurements

Cobalt oxide-coated *F. excelsior* SECs were used as an electrode active material for supercapacitor electrode production. While the Pt sheet electrode acts as the counter electrode, the leak-free electrode (Harvard Apparatus) acts as the reference electrode in a three-electrode cell. The electrochemical performance of the supercapacitor electrode was investigated by cyclic voltammetry (CV), electrochemical impedance spectroscopy (EIS), and a long-term galvanostatic charge–discharge (GCD) test using a Gamry Reference 3000 potentiostat/galvanostat/ZRA system.

## 3 Results and Discussion

### 3.1 Morphology

**Figure 1:**
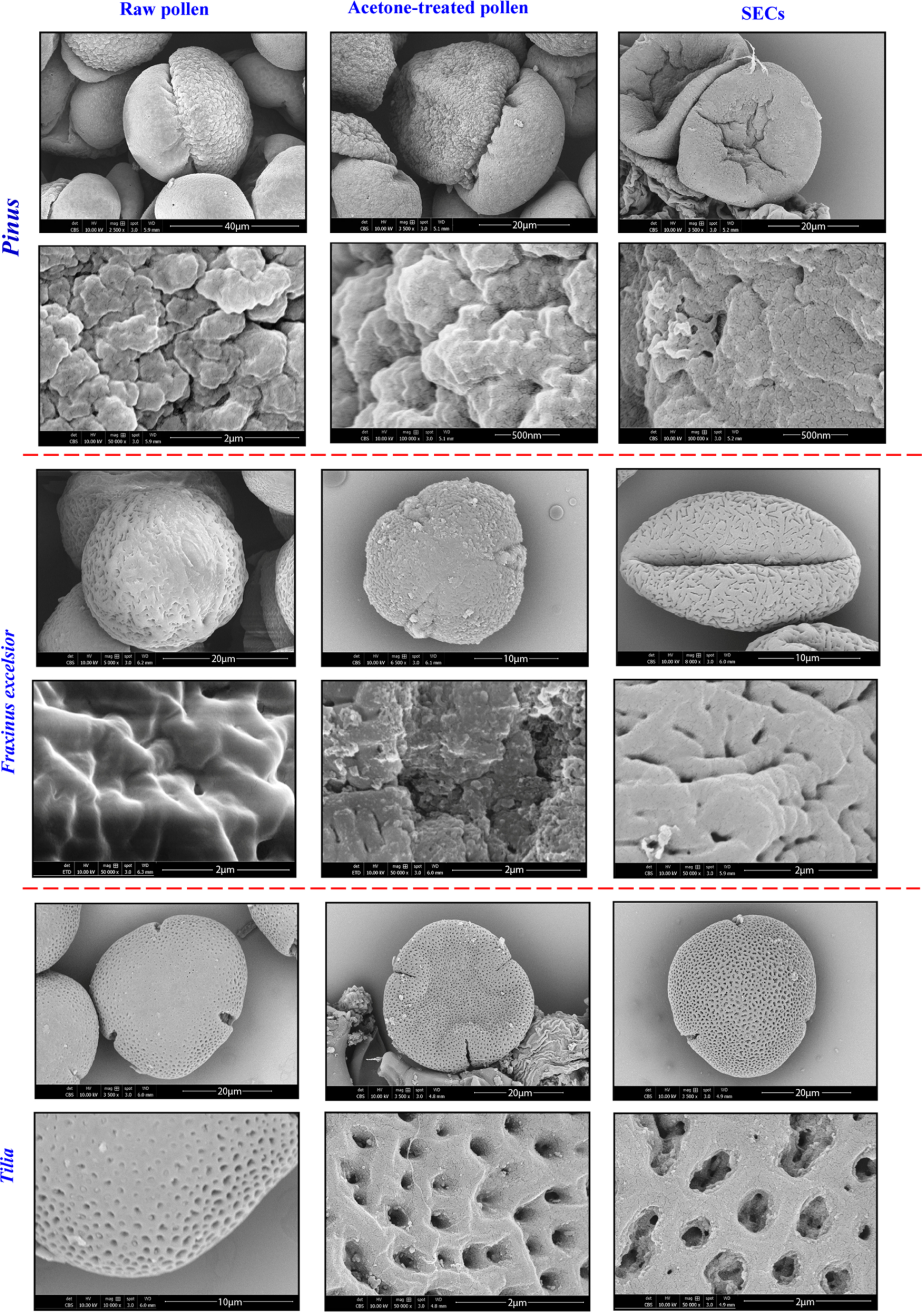
SEM images of raw pollen, acetone-treated pollen, and SECs at different magnifications.

The SEM images of raw pollen, acetone-treated pollen, and SECs are shown in Figure 1 at different magnifications. The morphological details of pollen were determined from SEM images; *Pinus* has a winged structure, *F. excelsior* has a reticulated surface, and *Tilia* has a perforated surface. The *Pinus* pollen grain has an elongated aperture (monosulcate) on the distal pole and two air sacs (bisaccate) for dispersal. The pollen body surface consists of irregular elements similar to warts (verrucate), and the sac surface is foveolate. The *F. excelsior* pollen grain is spheroidal and tricolpate. Its surface is beaded and netted reticulate. The *Tilia* pollen grain is tricolporate and the surface is perforated and reticulate. The ratio of the polar axis <length to equatorial diameter is <0.5 (peroblate) (Figure 1). The diameters of raw pollen and SECs were determined from SEM images (Table 1).

**Table 1:** Diameters, Surface Areas, Pore Volumes, and Pore Diameters of Raw Pollen and SECs

	raw pollen				SEC			
pollen	*D*_pollen_^a^ (μm)	*S*_BET_ (m^2^/g)	*V*_p_ (cm^3^/g) × 10^–3^	*D*_p_ (nm)	*D*_SEC_^a^ (μm)	*S*_BET_ (m^2^/g)	*V*_p_ (cm^3^/g) × 10^–3^	*D*_p_ (nm)
*Pinus*	40–43	2.4	1.919	4.9	30–39	1.17	0.554	3.6
*F. excelsior*	26–27				18–22	10.88	16.00	8.1
*Tilia*	34–37	1.37	1.048	4.5	34–35	1.05	1.232	6.3

a The diameters of raw pollen and SECs were measured based on SEM images. *D*_pollen_, diameter of raw pollen; *D*_SEC’_ diameter of SECs; *D*_p_, pore diameter; *S*_BET_, BET surface area; and *V*_p_, pore volume.

Morphological changes were observed in the surfaces of pollen grains after acetone and phosphoric acid treatments. The acetone treatment caused slight inflation of the cytoplasm and removed oils from the pollen surface, so the surface became rough; however, the internal structure and pores were not affected (Figure 1). The diameters of SECs shrunk by 17% in *Pinus*, 28% in *F. excelsior*, and 3% in *Tilia*.

**Figure 2:**
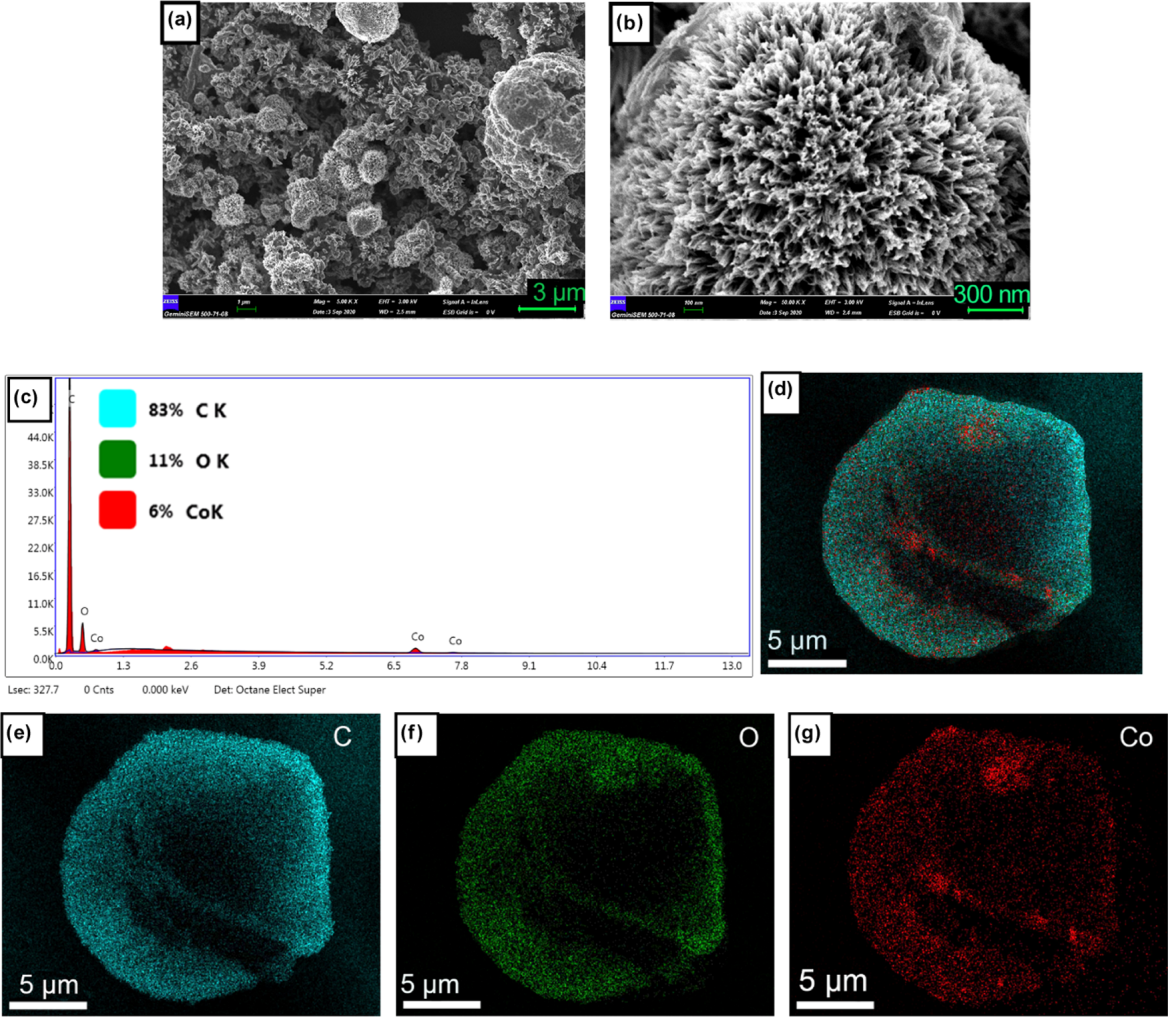
(a, b) SEM images at different magnifications and (c–g) elemental mapping by EDS of *F. excelsior* SECs coated with cobalt and then heat-treated at 360 °C (CoFSEC-360).

SEM images of *F. excelsior* SECs coated with cobalt are also given in Figure 2a,b. As can be seen from the figure, in addition to microsized granular structures on SECs, microspheres were formed with a diameter of about 1–4 μm, resembling a blue hedgehog thistle plant. The results of elemental mapping by energy dispersive X-ray spectrometer (EDS) show Co homogeneously distributed over the entire surface of the SECs (Figure 2g). EDS spectra also show that the atomic ratio of C/O/Co of the sample is 83:11:6 (Figure 2c).

### 3.2 Surface Areas, Pore Volumes, and Pore Diameters

The surface areas, pore volumes, and pore diameters of raw pollen, SECs, and cobalt-coated SECs were determined by the nitrogen adsorption–desorption isotherms. To determine how much a coating of the same material on different pollen SECs affects the surface area results, SECs of the three different pollen were reacted in the same hydrothermal bath conditions in a bath containing 25 mM cobalt ions and then heat-treated at 360 °C.

**Figure 3:**
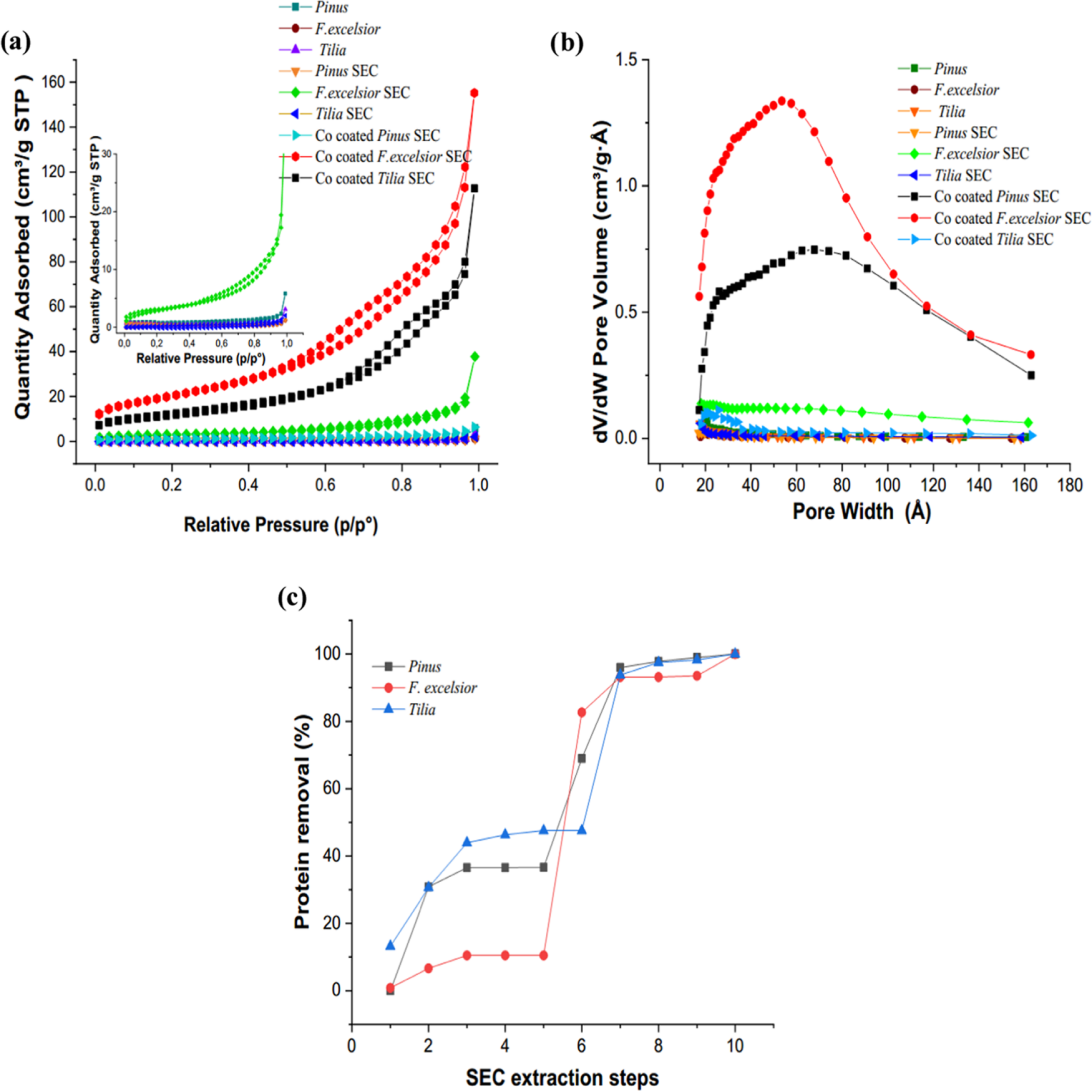
(a) N_2_ adsorption/desorption isotherms, (b) pore width distribution curves of raw pollen, SECs, and Co_3_O_4_-coated SECs, (c) protein quantification graphics of supernatants obtained from SEC extraction.

BET surface area measurements of the obtained samples are presented in Figure 3. Among the three different pollens, it can be seen in the *F. excelsior* pollen that there was a significant increase in both the surface area and the pore volume after the extraction process. However, no improvement was observed in *Pinus* pollen. The surface area and pore density of the materials formed on SECs with a higher surface area and high pore density were found to be high. The highest surface areas were obtained as 74.32 m^2^/g in the cobalt-coated sample on the *F. excelsior* SEC. Again, a partial increase in the surface area was observed in *Tilia* pollen after coating. In *Pinus* SECs and *Tilia* SECs, these values were determined to be 3.97 and 44.7 m^2^/g, respectively. Again, the highest pore volume density of 126.2 cm^3^/g was obtained in the structure grown on the *F. excelsior* SEC. The structures grown on *Tilia* were 4,398 × 10^–3^ cm^3^/g and those grown on *Pinus* SECs were 88.7 × 10^–3^ cm^3^/g. In raw *F. excelsior* pollen, the porosity of which could not be measured at the beginning, the average pollen diameter after extraction was 8.1 nm, while it was determined to be 5.68 nm after coating. This indicates that the pores of the *F. excelsior* pollen were well opened after extraction, and the cobalt coating formed on the SECs had a synergistic effect in improving the porosity.

### 3.3 Thermal Stability

**Figure 4:**
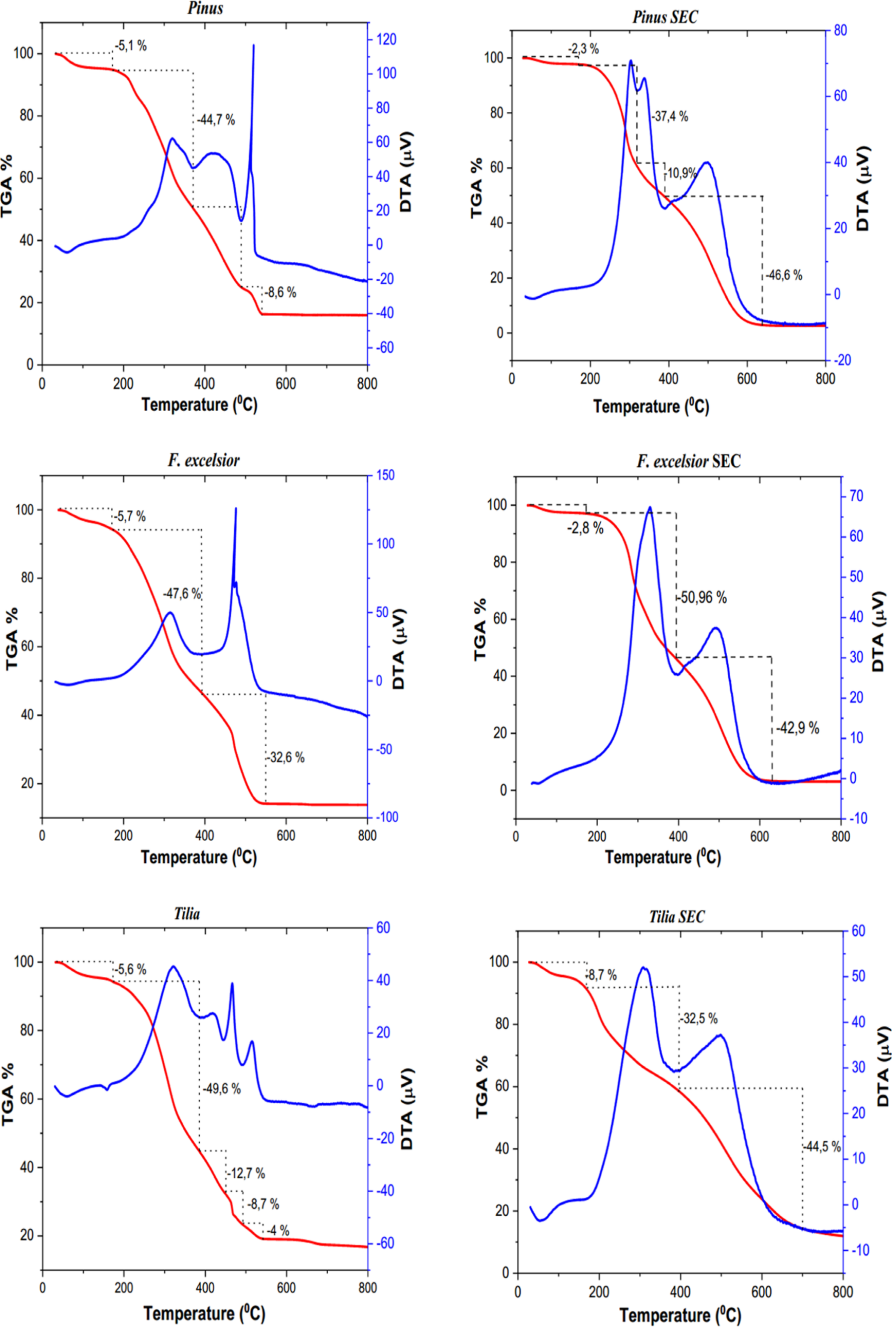
TGA/DTA thermograms of raw pollen and SECs.

Figure 4 shows the results of the thermogravimetric analysis of three different pollens and their SECs. The thermograms of raw *Pinus* pollen grains and their SECs consisted of four degradation steps, while raw *F. excelsior* pollen grains and their SECs consisted of three degradation steps. The raw *Tilia* pollen grain had five and its SEC had three degradation steps. This is because the very small holes in this raw *Tilia* pollen were more difficult to empty than those of the other two pollen. The extraction process took longer in *Tilia* than in *F. excelsior* pollen.

In general, the first mass loss seen in all raw pollen and SECs in the ∼30–172 °C range was related to the removal of adsorbed water and ethanol. The second and third mass losses were attributed to the degradation of carbohydrates, protein, and fatty acids, respectively, and the fourth mass loss was attributed to the degradation of the exine layer.^45^ The second mass loss was determined to be 44.6% in the temperature range of 173–372 °C in *Pinus* pollen, 47.6% in the temperature range of 172–391 °C in *F. excelsior* pollen, and 49.6% in the temperature range of 171–385 °C in *Tilia*. The third mass loss was 25.1% for *Pinus* in the temperature range of 372–489 °C, 32.5% for *F. excelsior* in the temperature range of 391–550 °C, and 12.7% for *Tilia* in the temperature range of 385–451 °C. The fourth mass loss of 8.6% between 489 and 540 °C occurred as a result of the thermal degradation of cellulose and the exine layer in *Pinus* pollen. In *Tilia*, the fourth and fifth mass losses were 8.7% in the temperature range of 451–492 °C and 4% in the temperature range of 492–538 °C, respectively.

The second mass loss was 37.3% in the 170–320 °C region in *Pinus* SECs and 50.9% in the 173–394 °C region in *F. excelsior* pollen. The third mass loss was 10.8% in the range of 320–390 °C in *Pinus* SECs, while it was 42.9% in the range of 394–630 °C in *F. excelsior* pollen. The fourth mass loss was 46.6% in the range of 390–640 °C in *Pinus* SECs, and it was not observed in *F. excelsior* SECs. Unlike raw *Tilia* pollen, *Tilia* SECs showed mass loss at three stages. The second mass loss of 32.5% occurred in the region of 169–394 °C. The third mass loss was 44.4% in the 394–700 °C region.

As a result of the extraction process, the final stage loss temperature was increased by approximately 100 °C in all SECs. This showed that the organic contents deteriorated by temperature were removed from the structure, and the desired biotemplate was left behind, which is more resistant to temperature and chemical processes. The highest mass loss of the exine capsule existed for the *Pinus* pollen and the longest degradation temperature interval was found in the *Tilia* SEC exine layer. We can say that all three pollen SECs were resistant to heat treatment below 400 °C.

To identify the thermal decomposition behavior of the CoFSEC microspheres, the TGA/DTG curves of neat FSEC and CoFSEC powders were used, as shown in Figure S1. In the case of FSEC powders, a main thermal decomposition was detected at the temperature range of 173–690 °C and the residue at 360 °C was above 63.5% (Figure S1a). For the thermally stabilized CoFSEC microspheres, thermal decomposition occurs dominantly at ∼279 °C (Figure S1b). The TGA/DTG curve also illustrates that the gradual decomposition of cobalt hydroxy carbonate to cobalt oxides and the degradation of the SECs occurred simultaneously in the temperature range of 289–429 °C, with a residue of ∼ 27.7 wt % (inset graph in Figure S1b).

### 3.4 Protein Removal

Samples obtained after each step of the SEC extraction process were used for protein quantification by measuring the absorbance at 280 nm. The acetone step was performed to remove the oil content of the pollen grains. The protein content was mostly removed by the orthophosphoric acid treatment (32.33% in *Pinus* and 72.21% in *F. excelsior*). However, in *Tilia* pollen grains, the removal was mostly in the first rinse with water after acidolysis (46.21%). Acetone and ethanol had little or no effects on protein removal. The protein content was not affected by acetone treatment in *Pinus* pollen and less affected in *F. excelsior* pollen (0.8%); however, this was affected in the *Tilia* pollen (13.22%) (Figure 3c).

### 3.5 C, H, and N Compositions

The removal of potentially allergic protein from natural materials used in vivo, such as for drug delivery, is very important. The presence of protein in pollen grains is directly proportional to the amount of nitrogen in the material. The lower the protein content, the less the intravenous drug will irritate the skin, while the oral drug will be less harmful. However, it is known that the oral administration of raw pollen has no allergen effect even in pollen-allergic patients.^46,47^

The total protein content in pollen is typically estimated from the total nitrogen content using a nitrogen-to-protein conversion factor. However, a limitation of this method is to accurately identify the conversion factor, which can vary from 5.18 to 6.38 among animal-based or plant-based protein sources. For pollens, there is no certain established conversion factor available. Nonetheless, a factor of 6.25 has been used in this study similar to that of Uddin et al.^48^ Another limitation of the nitrogen-to-protein conversion approach is that it may be overestimating the protein content. This is because the method assumes that all measured nitrogen comes from proteins; however, the nitrogen can also emanate from nonprotein compounds such as alkaloids.^48^ Therefore, the protein in the structure is expected to be much lower in real.^16^

CHN elemental analysis was performed to detect the effect of the new procedure on protein removal. The SECs of *Pinus* pollen grains had no nitrogen content after extraction. *F. excelsior* and *Tilia* SECs had a very low nitrogen content, indicating that the protein content was successfully removed (Table 2). Also, the highest hydrogen content was analyzed in the *F. excelsior* SECs. Results of CHN analysis indicate that the raw *Pinus* pollen grain contains 13.38% proteins, *F. excelsior* 35.06%, and *Tilia* 17.42%. The protein removal in *Pinus* SECs is 100%; ash tree 78,23%; and *Tilia* 53.71%. The protein content of SECs up to 8% indicates the successful protein removal and obtaining safe oral drug delivery materials free of allergenic protein. Additionally SEM, EDS, and FTIR measurements supported the conclusion that the pollen cytoplasm, which is the main source of nitrogen and contains protein, was removed after the extraction process.

**Table 2:** C, H, and N Compositions of Different Raw Pollens and SECs

	raw pollen				SEC			
pollen	C %	H %	N %	the protein content (%)	C %	H %	N %	the protein content (%)
*Pinus*	48.41	7.61	2.14	13.38	60.37	4.92		0.00
*F. excelsior*	48.61	7.39	5.61	35.06	59.52	7.41	1.22	7.63
*Tilia*	45.81	6.32	2.68	17.42	61.02	6.80	1.29	8.06

The nitrogen contents of SECs extracted with the new procedure show that all of the studied SECs are suitable for use in oral drug delivery. In contrast, it was noted that the high nitrogen content of the material enhances the capacitive properties of supercapacitor electrodes.^49^

### 3.6 Encapsulation Efficiency

The encapsulation efficiency varied according to the morphology and the loading method. BSA was better loaded into SECs by the centrifuge loading method. *Tilia* SECs were the most efficient SECs in the centrifuge loading method. For the passive loading method, *F. excelsior* SECs had the best loading capacity (Table 3).

**Table 3:** Encapsulation Efficiency of Different Pollen SECs

		amount of BSA in BSA-loaded SECs (mg)		loading content (%)		encapsulation efficiency (%)	
pollen	theoretical loading content (%)	passive loading	centrifuge loading	passive loading	centrifuge loading	passive loading	centrifuge loading
*Pinus*	130	0.31	0.51	3.1	5.1	2.39	3.92
*F. excelsior*	130	0.79	1.01	7.9	10.1	6.08	7.77
*Tilia*	130	0.76	1.33	7.6	13.3	5.85	10.23

### 3.7 FTIR Spectra

FTIR spectra of raw pollen and their SECs are shown in Figure S2. FTIR analysis confirmed the removal of proteins from pollen shells. The vibration peaks of 1640, 1550, and 1231 cm^–1^ belonging to proteins in the raw pollen decreased after the extraction process. The first peak belongs to the C=O stretching in amide I, the second peak belongs to the N–H bending and C–N stretching in amide II, and the last peak belongs to the N–H bending, C–N, and C–C stretching in amide III.

The chemical interactions between BSA and SECs were also analyzed by FTIR spectra (Figure 4). In the spectrum of BSA, O–H stretching vibrations were observed at 3257 cm^–1^. The C–H absorption band at 2958 cm^–1^ represented the CH_3_ group of proteins, and the peak that appeared at 1645 cm^–1^ showed the carbonyl compounds of amide I. The aromatic rings of amino acids were detected at 1511 cm^–1^ and at 1391 cm^–1^ in the fingerprint region.

**Figure 5:**
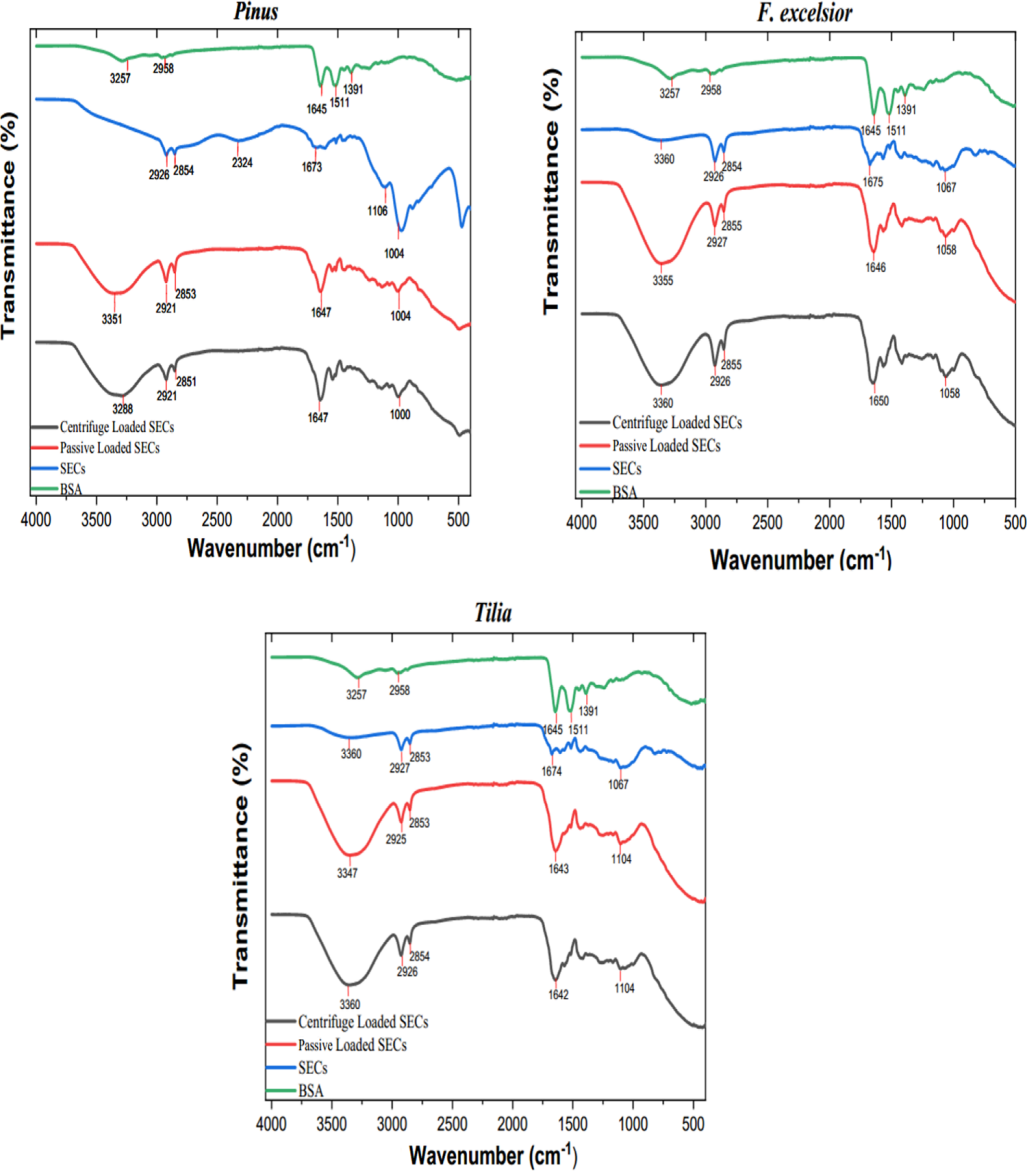
FTIR spectra of BSA, SECs, passive-loaded BSA, and centrifuge-loaded BSA, for three different SECs.

The broad peaks at 3305 cm^–1^ in *Tilia* SECs and 3360 cm^–1^ in *F. excelsior* SECs were associated with the O–H stretching. Aliphatic C–H stretching vibrations were observed at 2926–2921 and 2853–2855 cm^–1^ with two close peaks in all SECs (Figure 5). The triple bond (C≡C) vibration was noted at 2324 cm^–1^ in *Pinus* SECs. The peaks at 1675–1673 cm^–1^ indicate the C=C stretching. The aromatic rings were seen at 1106–1067 cm^–1^.

After BSA loading, the depth of the broad peaks was greater at 3360–3288 cm^–1^, representing the O–H absorption bands and indicating chemical interactions between BSA and SECs. The other changes in the spectrum were increases in band absorbances at 1650–1643 cm^–1^ and decreases at 1106–1000 cm^–1^ due to the overlapping with BSA. The absorption band at 2324 cm^–1^ disappeared after encapsulation in *Pinus* SECs. The FTIR spectra indicate successful encapsulation.

The effect of both the hydrothermal reaction in the bath containing Co ions and the heat treatment at 360 °C on *F. excelsior* SECs can be clearly seen from the FTIR spectrum in Figure S3. *F. excelsior* SECs and their heat treatment are named FSEC and FSEC-360, respectively. Again, in the bath containing Co ions, the hydrothermally reacted FSEC was named CoFSEC, and then the same sample, which was also heat-treated at 360, was named CoFSEC-360. In both FSEC and CoFSEC, the peaks of the aliphatic C–H stretching vibrations at 2926 and 2855 cm^–1^ and the peak of the aromatic C–H band at 831 cm^–1^ disappeared after heat treatments, and the SEC polymeric structure was oxidized. The band around 1677 cm^–1^ belonging to the FSEC phenolic ring shifted to 1724 cm^–1^ as a broader band, which also indicates that the polymer structure is oxidized. Oxidation causes aromatic peaks to increase in size and move into the higher wavenumbers.^50^ Also, some new peaks that appeared at 1513, 1417, 1067, and 831 cm^–1^ in the CoFSEC sample can be related to cobalt hydroxyl carbonate.^51^ The strong absorption peak observed around 656 cm^–1^ in the CoFSEC-360 sample confirms the formation of the Co_3_O_4_ structure.

### 3.8 Electrochemical Capacitive Performance of SECs Based Co_3_O_4_ Electrode

**Figure 6:**
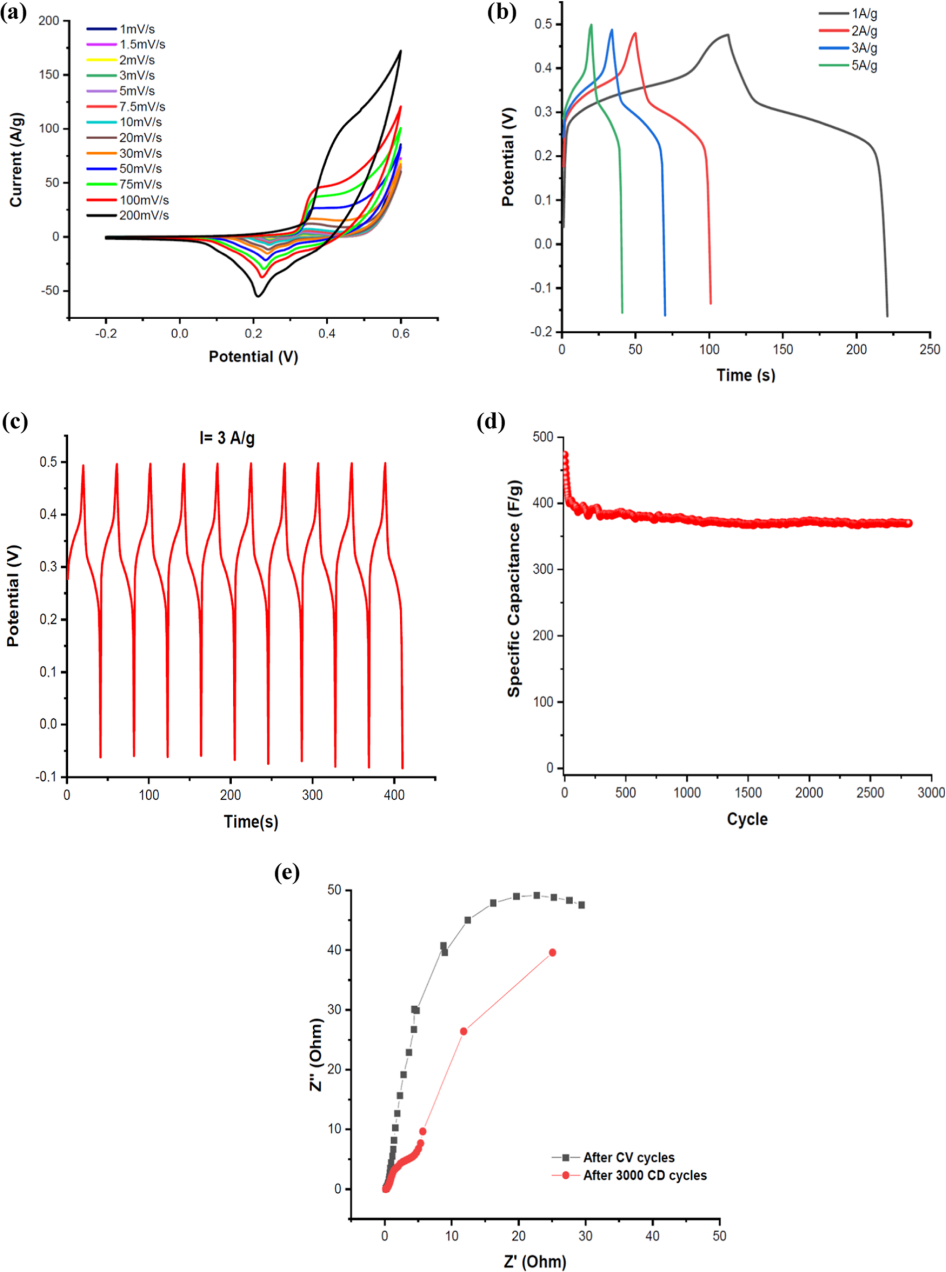
Electrochemical capacitive properties of *F. excelsior* based Co_3_O_4_ the electrode in 6M KOH solution. (a) CV curves at different scan rates, (b) CD behaviors at different current densities, (c) ten cyclic GDC curves. (d) variation of the specific capacitance as a function of cycle number. The discharge current is 3 A/g and (e) Nyquist plots at the open-circuit potential.

CV measurements of the Co_3_O_4_-SEC electrode in the −0.2 and +0.6 V potential regions, at scanning speeds of 1–200 mV/s, are given in Figure 6a. In the three-electrode cell, 6M KOH was used as the electrolyte. The best electrochemical performance was observed in the 360 C heat-treated sample. The dominance of pseudocapacitive behavior resulting from faradic reactions occurring on the electrode surface was observed in the CV curves. In addition, an increase in peak current values was observed with an increasing scanning speed. This shows that the supercapacitor electrode responds quickly to fast charge–discharge tests.

CD tests at different current densities were also performed in a three-electrode cell (Figure 6b,c). In addition, long-term and consecutive (at least 3000) charge–discharges of the formed electrode are presented in Figure 6d. Specific capacitance values were calculated from the GCD cycles using Formula 3.
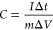
3where *I* is the current, *m* is the mass of the active material, Δ*t* is the time taken to discharge, and Δ*V* is the potential difference during the discharge. The specific capacitance of the Co_3_O_4_ electrode containing *F. excelsior* SECs, at a constant current value of 3 A/g, was initially 473 F/g, but this value decreased rapidly to 380 F/g after 165 cycles and was calculated to be a minimum of 373 F/g at the end of the 3000th cycle.

After CV measurements and after 3000 continuous GCD measurements, EIS measurements were carried out in the frequency range from 100 kHz to 5 MHz with 10 frequencies per decade, at the open-circuit potential and applying a 5 mA perturbation amplitude. The slope of the linear line in the high-frequency region decreased from 71 to 54° after 3000 consecutive GCDs. In a supercapacitor, this value should be at least 45°. The results show that the *F. excelsior* SEC-containing Co_3_O_4_ material is a good candidate for supercapacitor electrode design. It was observed that the electrode contact resistance maintained its initial value even after GCD cycles (Figure 6e).

## 4 Conclusions

In this study, research was conducted on the multifunctional use of SECs obtained from different tree pollens in drug delivery studies and supercapacitor studies.

First, we have produced porous, environmentally friendly, and economical biotemplate polymers from ubiquitous tree pollen by a simple acidolysis method. A new method was applied successfully for protein removal in pollen grains with low protein content. After acetone treatment, the structural integrity was preserved, and the number and the width of pores had increased but the surface remained undamaged. Also, the full pores of raw pollen were evacuated after orthophosphoric acid treatment. The 30 min ultrasonic process applied during both treatments reduced the time required for protein removal from sporopollenin, which was reported in previous studies, from 36 h to a total of 60 min. We produced ubiquitous, porous, environmentally friendly, and economical biotemplate polymers from pollen by a simple acidolysis method. The protein, carbohydrate, and fat content of pollen grains demonstrate differences between the species. For this reason, there were differences between the degradation steps of pollen grains and the interval of the steps.

The efficiency of the loading capacity was investigated for SECs of three different morphologies. It was found that the surface morphology and porosity affect the drug loading capacity. While the large pore and netted surface in *F. excelsior* SECs limited the loading of BSA by centrifuge loading, the narrow pore and verrucous surface in *Pinus* SECs made it difficult to load drugs by passive and centrifuge loading. The perforated surface in Tilia SECs allowed the best encapsulation efficiency by centrifuge loading.

Second, these three different SECs were coated with cobalt oxides to increase their surface area. It has been shown that the Co_3_O_4_-SEC structure can be used as the active material in supercapacitor electrodes. The carbon and hydrogen content of SECs allow the growth of porous metal oxide structures with a higher surface area. Additionally, the nitrogen content is not an obstacle for producing a supercapacitor electrode; on the contrary, it improves capacitive properties. In addition, the exine layer generally begins to deteriorate after 400 °C and therefore these SECs can be used in the production of supercapacitor electrodes based on metal oxides such as NiO and Co_3_O_4_. Although the surface area and pore density parameters of both raw pollen and SECs were initially very low, they increased significantly with cobalt oxide coating and the subsequent thermal process.

The increase in the porosity of the SECs obtained from the *F. excelsior* pollens is seen from both SEM images and BET results. After the hydrothermal reaction with cobalt and urea, it was determined by SEM and elemental mapping measurements that a homogeneously distributed cobalt coating was present on the SEC surfaces. The Co_3_O_4_ structure was obtained after the heat treatment applied at 360 °C. The increase in porosity is determined by BET measurements, after the hydrothermal process, and after the heat treatment. The use of both a porous biotemplate and nanostructures grown on this template, which has pseudocapacitive properties, has created a synergic effect.

In particular, the surface area of *F. excelsior* covered with Co_3_O_4_ increased to 74.32 m^2^/g. The capacitance value of the supercapacitor electrode obtained using this material as an active material was 473 F/g. This value was reduced to 380 F/g after 165 cycles and then remained at 98% after 4835 cycles at a constant 3A/g.

## Supporting Information Available

SEC extraction steps (page S2); thermal decomposition behavior of the FSEC and CoFSEC microspheres (Figure S1); FTIR spectra of raw pollen and their SECs (Figure S2); and FTIR spectra of *F. excelsior* SECs after hydrothermal reaction and after heat treatment (Figure S3) (PDF)
