# Supplementary material for: Different Plant Sporopollenin Exine Capsules and Their Multifunctional Usage
Source: ACS Appl Bio Mater. 2022 Feb 24;5(3):1348–60. doi: 10.1021/acsabm.2c00071 (PMC8941510; doi:10.1021/acsabm.2c00071)
Supplement: Supplementary file 1 — mt2c00071_si_001.pdf [file mt2c00071_si_001.pdf]

Supporting Information

Different plant sporopollenin exine capsules and their multifunctional usages

Funda Ersoy Atalay[a,*], Ayse Asiye Culum[b], Harun Kaya[c], Gunay Gokturk[a], Emel Yigit[d]

[a]Inonu University, The Faculty of Science and Arts, Department of Physics, 44280, Malatya, Turkey

[b]Malatya Turgut Ozal University, Vocational School of Health Services, Department of Medical Services and Techniques, 44210, Malatya Turkey

[c]Malatya Turgut Ozal University, Faculty of Engineering and Natural Sciences, 44210, Malatya, Turkey

[d]Inonu University, The Faculty of Science and Arts, Department of Biology, 44280, Malatya, Turkey

[*]Corresponding author : Funda Ersoy Atalay (e-mail: funda.atalay@inonu.edu.tr)

Number of pages: 5; Number of figures:3

The SEC extraction steps

2 μL of the supernatant obtained after each step of the SEC extraction process were used for protein determination and analyzed for absorbance at 280 nm by Take3 plate of the spectrophotometer (BioTec Epoch). The supernatant collection steps are numbered as follows:

- Step 1: After acetone treatment,
- Step 2: The first rinse with pure water after acetone treatment,
- Step 3: The second rinse with the pure water after acetone treatment,
- Step 4: The first rinse with the ethanol after acetone treatment,
- Step 5: The second rinse with ethanol after acetone treatment,
- Step 6: After orthophosphoric acid treatment,
- Step 7: The first rinse with pure water after orthophosphoric acid treatment,
- Step 8: The second rinse with the pure water after orthophosphoric acid treatment,
- Step 9: The first rinse with the ethanol after orthophosphoric acid treatment,
- Step 10: The second rinse with ethanol after orthophosphoric acid treatment.

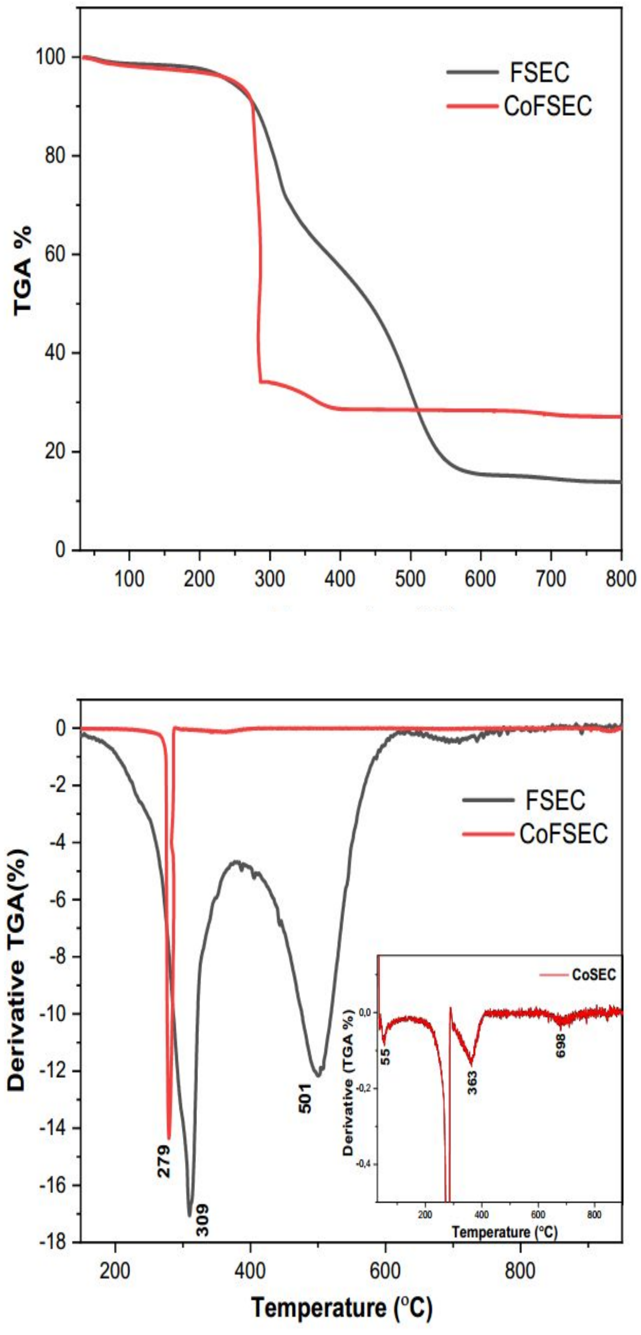

Figure S1: Thermal decomposition behavior of the FSEC and CoFSEC microspheres.

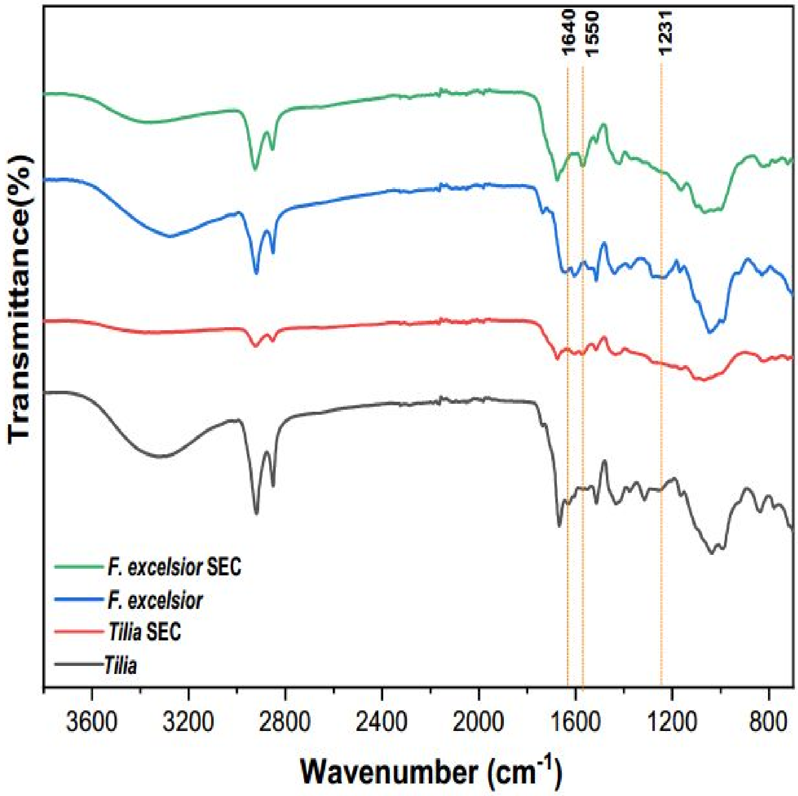

Figure S2: FTIR spectra of raw pollen and their SECs

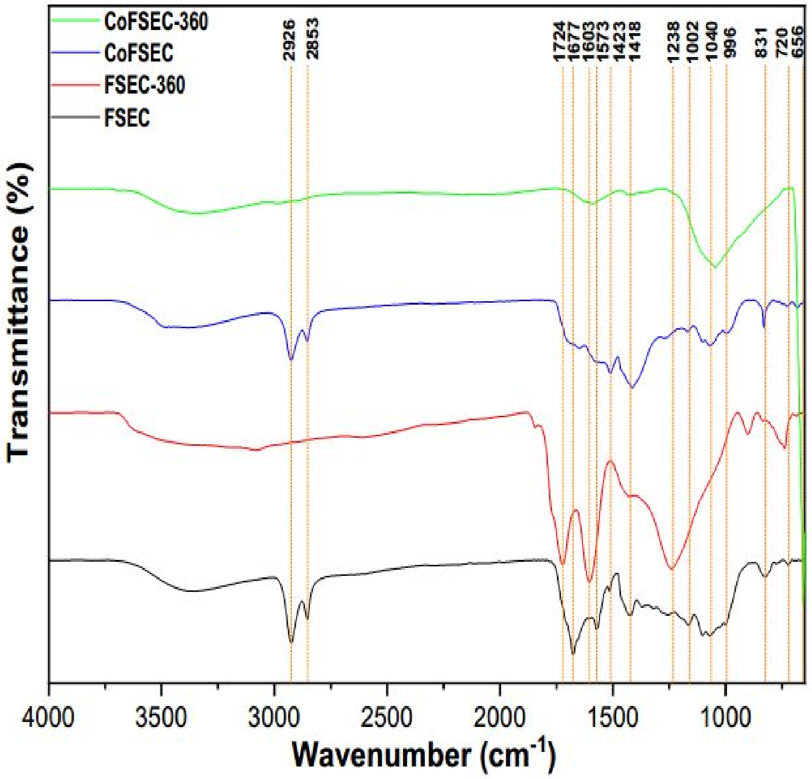

Figure S3: FTIR spectra of *F. excelsior* SECs after hydrothermal reaction and after heat treatment.
